# Cleaning interfaces in layered materials heterostructures

**DOI:** 10.1038/s41467-018-07558-3

**Published:** 2018-12-19

**Authors:** D. G. Purdie, N. M. Pugno, T. Taniguchi, K. Watanabe, A. C. Ferrari, A. Lombardo

**Affiliations:** 1 Cambridge Graphene Centre, University of Cambridge, 9 JJ Thomson Avenue, Cambridge, CB3 0FA UK; 2 Laboratory of Bio-inspired and Graphene Nanomechanics, Department of Civil, Environmental and Mechanical Engineering, University of Trento, via Mesiano, 77, I-38123 Trento, Italy; 3 School of Engineering and Materials Science, Queen Mary University of London, Mile End Road, London, E1 4NS UK; 4 Ket-lab, E. Amaldi Foundation, Via del Politecnico, 00133 Rome, Italy; 5 National Institute for Materials Science, 1-1 Namiki, Tsukuba, 305-0044 Japan

## Abstract

Heterostructures formed by stacking layered materials require atomically clean interfaces. However, contaminants are usually trapped between the layers, aggregating into randomly located blisters, incompatible with scalable fabrication processes. Here we report a process to remove blisters from fully formed heterostructures. Our method is over an order of magnitude faster than those previously reported and allows multiple interfaces to be cleaned simultaneously. We fabricate blister-free regions of graphene encapsulated in hexagonal boron nitride with an area ~ 5000 μm^2^, achieving mobilities up to 180,000 cm^2^ V^−1^ s^−1^ at room temperature, and 1.8 × 10^6^ cm^2^ V^−1^ s^−1^ at 9 K. We also assemble heterostructures using graphene intentionally exposed to polymers and solvents. After cleaning, these samples reach similar mobilities. This demonstrates that exposure of graphene to process-related contaminants is compatible with the realization of high mobility samples, paving the way to the development of wafer-scale processes for the integration of layered materials in (opto)electronic devices.

## Abstract (web summary)

Atomically-clean interfaces are required in heterostructures. Here, authors report a method for fast and parallel removal of contaminants from fully-formed heterostructures, including sample intentionally exposed to polymers and solvent, achieving room temperature mobility over 180,000 cm^2^/Vs for graphene.

## Introduction

The process of creating materials with pre-determined properties has been one of the key element of success of modern solid-state electronics and opto-electronics. Heterostructures, i.e., heterogeneous structures built by combining two or more different materials, were introduced in the fifties^1,2^, enabling the engineering of complex structures with tailored properties such as superlattices^3^. Semiconductor-based heterostructures play a major role in modern integrated electronics and optoelectronics, enabling applications such as solid-state lasers^4^, high electron mobility transistors^5^ and quantum cascade lasers^6^.

More recently, another class of materials by design has arisen due to the possibility of stacking single layer graphene (SLG) and other layered materials into heterostructures^7–13^. By varying the layered materials used, and the angle between them^14,15^, this gives rise to a virtually infinite set of options for creating different heterostructures, not previously produced in the field of semiconductor based super-lattices. However, a number of challenges remain before such heterostructures can be widely applied, such as the need to use layered materials prepared by scalable techniques, like chemical vapor deposition (CVD)^16,17^, and to achieve clean interfaces over the entire heterostructure.

The most widely studied layered material heterostructure is SLG encapsulated in hexagonal boron nitride (hBN)^18–24^. Room temperature (RT) charge carrier mobility (*μ*) in hBN-encapsulated SLG can reach values over an order of magnitude higher than SLG on SiO_2_^18,19^. Furthermore, encapsulation isolates SLG from sources of contamination, such as lithographic polymers and solvents used during device processing^19^, or ambient air^20^, which can otherwise degrade mobility^22,25^ and increase doping^20^. Thus, hBN encapsulated SLG could enable state of the art performance for a range of applications in high-frequency electronics^13,26,27^ and (opto)electronics^28,29^.

Encapsulated SLG and other layered material heterostructures are assembled by first producing the individual layered materials on separate substrates, typically Si + SiO_2_^19^, or polymers, such as Polymethyl methacrylate (PMMA)^18,30^, followed by transfer and stacking to achieve the desired heterostructure^18,19,30^. During stacking, contaminants such as hydrocarbons^31^, air^23^, or water^32,33^, can become trapped between the layers, aggregating into spatially localized pockets with typical lateral sizes from a few nanometers^34^ up to micrometers^23^, known as blisters^23^ or bubbles^19,20,31^, which form due to the interplay of the layered material elastic properties and van der Waals forces^34^. This aggregation of contaminants into blisters leaves the regions located between them with clean interfaces^31^, and devices can therefore be fabricated exploiting these areas^30^. However, the device size is constrained by the blister spacing, typically 1–10 μm^30^. It is therefore paramount to develop cleaning techniques capable of removing blisters over the entire dimensions of a heterostructure.

Blister-free areas >10 μm can be obtained by using a hot pick-up technique^23^, where adsorbates present on the layered material surface can be removed during encapsulation by bringing the layers together in a conformal manner at 110 °C^23^. The cleaning in this process is due to higher diffusivity of the contaminants at 110 °C than at room temperature^23^, allowing them to diffuse out of the sample during encapsulation. Blister-free regions were also reported in ref. ^19^, although no explanation of how blisters are avoided was given. In refs. ^19,23^ residual blisters sometimes remained within the heterostructure due to incomplete cleaning during transfer, which could not then be removed. Furthermore, the technique of ref. ^23^ only achieves clean interfaces when the encapsulation is performed slowly, with lateral speeds <1 μm s^−1^. The required cleaning time would further scale with the total number of interfaces within the heterostructure. Therefore, while refs. ^19,23^ are in principle capable of cleaning interfaces over areas larger than the ~20 μm reported to date^19,23^, their suitability to cleaning wafer-scale sized samples is limited. There is therefore a critical need to develop techniques allowing rapid, parallel (i.e., independent from the number of layers forming the heterostructure) and repeatable assembly and cleaning of heterostructures. Ref. ^23^ also produced heterostructures using SLG intentionally contaminated with PMMA residuals left from lithographic processing, suggesting that the hot pick-up technique could be used to exclude these polymer residuals. However, no comparison was given of the mobility of samples produced using clean and polymer contaminated SLG.

Here we show how to remove contamination trapped within already assembled heterostructures. This is achieved by laminating the heterostructure onto a SiO_2_ substrate at ~180 °C. At this temperature the blisters become physically mobile, enabling them (and the contaminants trapped inside) to be pushed to the sample edges, where they are eliminated. We achieve blister-free hBN-encapsulated SLG with areas up to ~5000 μm^2^, limited only by the size of the exfoliated flakes. We manipulate blisters at speeds >10 μm s^−1^, over an order of magnitude faster than ref. ^23^. Our approach also allows the heterostructure interfaces to be cleaned simultaneously, unlike existing techniques, where the interfaces need to be cleaned sequentially^19,23^. Furthermore, our cleaning method also works for heterostructures based on different materials, such as hBN/MoS_2_ and hBN/SLG/MoS_2_, indicating the general suitability of our approach.

We fabricate hBN/SLG/hBN Hall bars with widths *W* up to 24 μm achieving mobilities up to 180,000 cm^2^ V^−1^ s^−1^ at room temperature. The mobility is consistently high across all samples, with an average ~160,000 cm^2^ V^−1^ s^−1^ across 15 Hall bars. We also report mobilities up to ~1.8 × 10^6^ cm^2^ V^−1^ s^−1^ at 9 K. Moreover, we show that our approach works on SLG intentionally exposed to PMMA, acetone and isopropyl alcohol (IPA) before encapsulation, achieving mobilities up to ~150,000 cm^2^ V^−1^ s^−1^ at room temperature after cleaning, i.e., there is no mobility degradation compared to non-contaminated SLG. We show micro-meter scale ballistic transport in our initially contaminated SLG through bend-resistance measurements, therefore demonstrating that with appropriate cleaning the mobility of polymer and solvent contaminated SLG can be equivalent to the highest quality encapsulated samples in which the interfaces are clean^8,19,20,30^. The mobility we achieve is around an order of magnitude higher than in other polymer and solvent contaminated SLG/hBN samples reported in literature^18,30^. Our approach paves the way to the optimization of scalable techniques, such as wet^35^ and (or) polymer assisted transfers^36,37^, for the fabrication process of high mobility encapsulated SLG and other heterostructures.

## Results

### Encapsulation, cleaning, and device fabrication

**Fig. 1:**
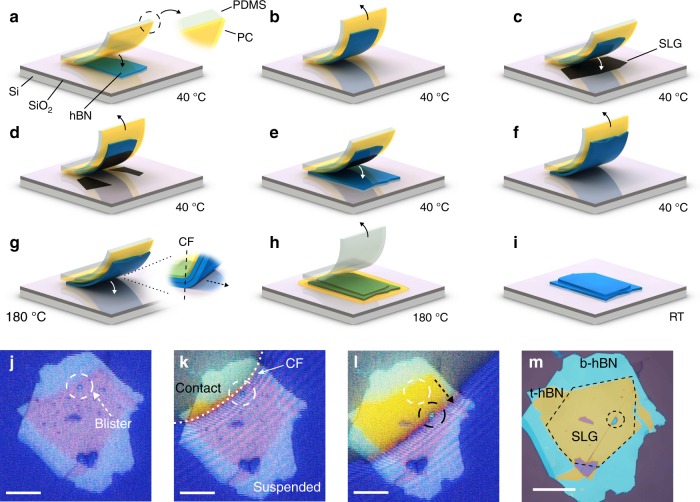
Scheme of the cleaning process. **a** A stamp, consisting of a PC film (yellow) mounted on a PDMS block (white-translucent) is brought into contact with a hBN flake (blue) exfoliated on SiO_2_ + Si (purple/light gray). **b** The stamp is withdrawn, picking up the hBN. **c** The hBN is lowered into contact with an exfoliated SLG (black), and then withdrawn, **d** picking the SLG portion in contact with hBN. **e** hBN and SLG are brought into contact with another hBN flake, forming the encapsulated stack. **f** Encapsulated stack is picked up from the SiO_2_ + Si substrate. Steps **a**–**f** are performed at 40 °C. **g** The temperature is raised to 180 °C and the encapsulated stack is laminated onto SiO_2_ + Si. The contact front (CF) is defined as the interface between the portion of the heterostructure suspended and that in contact with the SiO_2_ + Si. Control over the stamp height determines the CF lateral movement. This is achieved by tilting the PDMS block, such that the stamp first contacts the substrate on one side. As the CF encounters blisters, these are manipulated and removed. **h** The stamp is withdrawn. The PC adheres to the substrate, PDMS is peeled away. **i** PC is dissolved in chloroform. **j**–**m**: optical images of the process. **j** Encapsulated sample suspended on the PC stamp above Si + SiO_2_. One blister is highlighted with a dashed white circle. Other blisters are seen as dark spots. **k** The sample is laminated onto Si + SiO_2_. The CF between PC and substrate is marked with a white dashed line. Above the CF, the PC is in contact with SiO_2_, while below it is suspended. **l** as the CF advances it pushes the blisters. The blister in j, originally in the position marked by the white circle, has now moved, as marked by the black circle. The arrow shows the direction of movement. **m** hBN/graphene/hBN heterostructure after removal of PC. The dashed black line marks the SLG location. Scale bars 20 μm

Figure 1 shows a schematic representation of our approach to produce hBN/SLG/hBN heterostructures. Flakes of hBN and SLG are prepared by micro-mechanical cleavage (MC)^38^ on Si + 285 nm SiO_2_ (see Methods). Suitable SLG and hBN flakes are identified prior to transfer by a combination of optical microscopy^39^ and Raman spectroscopy^40–43^. We fabricate heterostructures with a range of hBN thicknesses, *t*_hBN_ (2–300 nm), and widths, *W*_hBN_ (up to ~200 μm), observing blister manipulation and cleaning in all cases.

In order to pick up and transfer the flakes we use a stamp consisting of a layer of polycarbonate (PC) mounted on a block of polydimethylsiloxane (PDMS) for mechanical support, Fig. 1a. The stamp is similar to that used in ref. ^19^, however we use PC instead of poly-propylene carbonate (PCC) as our cleaning requires a temperature of ~180 °C, well above the PPC glass transition *T*_g_ ≈ 40 °C^44^.

The stamp is placed on a glass slide attached to a micro-manipulator (resolution ~1 μm) under a microscope. The Si + SiO_2_ substrates, with the flakes to be transferred, are positioned underneath the micro-manipulator, on a heated stage, enabling temperature control from room temperature up to 300 °C.

The process begins by placing the PC into contact with a selected hBN flake, then withdrawing, while keeping the substrate at 40 °C, Fig. 1a. This temperature is chosen because it allows us to pick both hBN and SLG flakes with a success rate ~ 100% (as compared to room temperature, where this is <90%). The hBN adheres to the PC surface and is removed from the Si + SiO_2_ as the stamp is lifted, Fig. 1b. We then position the hBN over a chosen SLG flake and bring the two into contact, before again withdrawing while still at 40 °C. The portion of the SLG in contact with hBN delaminates from the Si + SiO_2_, while that in contact with the PC remains on the Si + SiO_2_, due to the preferential adhesion of SLG to hBN^23^ Fig. 1c, d. hBN and SLG flakes are then aligned and brought into contact with another (bottom) hBN flake, Fig. 1e, encapsulating the SLG.

We next withdraw the stamp with the heterostructure still attached to the PC, suspending it above the Si + SiO_2_, Fig. 1f. The stage temperature is increased to 180 °C, following which the stamp is brought into contact with the substrate, Fig. 1g. During this step the PDMS block is tilted ~1°, so that contact with the substrate first occurs on one side of the stamp, and then advances horizontally across it. Control over the stamp vertical position also defines the position of the contract front (CF) in the horizontal direction. The CF is the interface between the portion of the stamp in contact with Si + SiO_2_, and that suspended, as in Fig. 1g. At 180 °C the PC is above *T*_g_ ~ 150 °C^45^, resulting in decreased viscosity^46^, allowing greater control over its lateral movement. Below *T*_g_, the CF can move laterally in uncontrolled, discrete jumps.

As the CF approaches the encapsulated SLG, we observe the aggregation of numerous blisters, Fig. 1j. An example of typical blister coverage is reported in Supplementary Fig. 1. We attribute this to the heterostructure approaching the Si + SiO_2_ surface, resulting in its temperature increasing to ~180 °C. At room temperature, trapped contaminants cover the sample interfaces^23^, but become increasingly mobile, segregating into spatially localized blisters as the temperature rises above ~70 °C^23^.

When the CF passes across the encapsulated stack, the stack is laminated onto the Si + SiO_2_, Fig. 1g. This pushes any blisters within the heterostructure in the direction of the advancing CF (see Supplementary Movies 1 and 2). As blisters are swept through the heterostructure they collide and aggregate. They continue to move until they reach the heterostructure edge, at which point they are eliminated, or until they reach a physical discontinuity, such as a crack or wrinkle in the hBN or SLG, which may pin them. Once the CF has fully passed across the encapsulated stack and the blister removal is complete, the stamp is withdrawn, Fig. 1h. At 180 °C the PC preferentially adheres to the SiO_2_, allowing the PDMS to be peeled away, leaving the PC adhered to the SiO_2_ + Si surface, Fig. 1h. The PC is then removed by rinsing the sample in chloroform for ~10 min, Fig. 1i. In the hot-pick up method a 15 min bake at 130 °C is used to promote adhesion between the stack and substrate following transfer^23^. Here no post transfer bake is necessary, as the 180 °C used during transfer (maintained for ~2–3 min to allow the PC to melt) is sufficient to promote adhesion between stack and substrate.

**Fig. 2:**
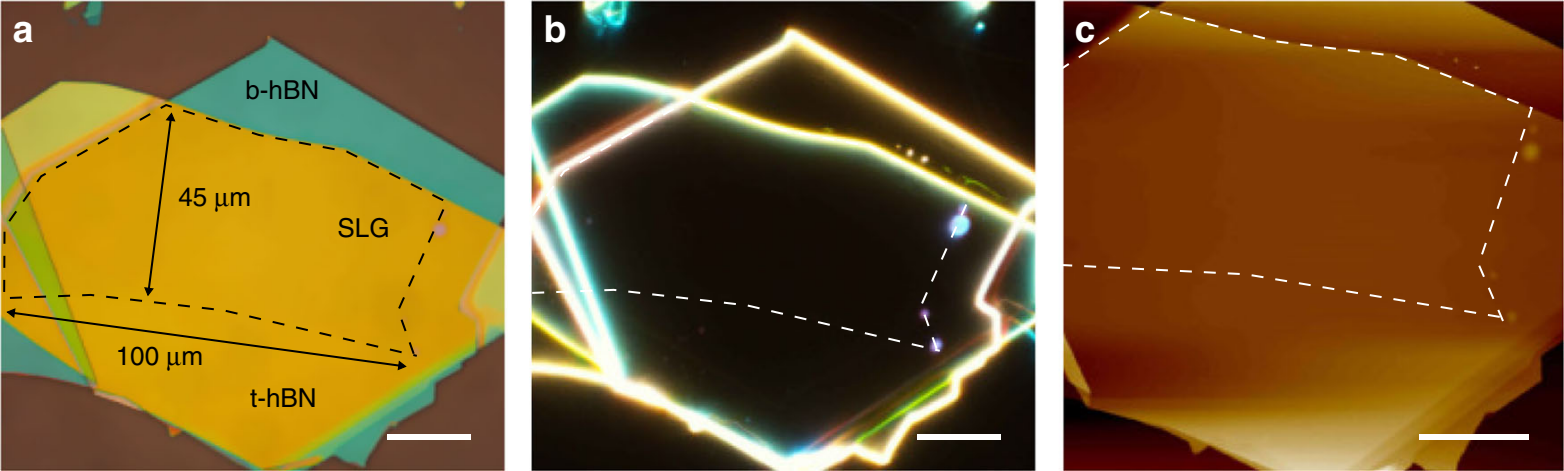
Optical images and AFM scans of a heterostructure after cleaning. **a** Optical t-hBN: top hBN. b-hBN: bottom hBN. SLG: single layer graphene. **b** Optical dark field image of the sample. **c** AFM. The dashed lines show the location of the SLG within the heterostructure. Scale bars 20 μm

Figure 1j–l show the movement of blisters in response to the advancing CF. Figure 1j is the sample before cleaning, suspended on the PC stamp above Si + SiO_2_. Numerous blisters can be seen. In Fig. 1k the CF (marked by the white dashed line) is advancing across the heterostructure. Above the CF (yellow optical contrast) the PC is in contact with Si + SiO_2_. In Fig. 1l the CF has advanced further. One blister is highlighted, with its initial location marked by a dashed white circle in Fig. 1j–l, and by a dashed black circle in Fig. 1l after being moved by the advancing CF. Figure 1m is the same sample after cleaning. One blister (highlighted by a dashed black circle) remains, pinned by a wrinkle. A second heterostructure also encapsulated and cleaned using the same method is shown in Fig. 2a, with optical dark field shown in Fig. 2b, and atomic-force microscopy scan in Fig. 2c. This is blister-free over ~100 μm × 45 μm. Further examples of hBN/SLG/hBN heterostructures are shown in Supplementary Fig. 2.

Blisters are manipulated at speeds >10 μm s^−1^. They can also be pulled by withdrawing instead of advancing the CF, i.e., they can be continuously manipulated both forwards and backwards (see Supplementary Movie 3). We find no effect of the tilt angle on the cleaning process for angles in the range ~0.5–5°. The presence of SLG in the heterostructure plays a significant role in the ability to manipulate the blisters using the CF. Blisters are always manipulated by the CF in the hBN/SLG/hBN portion of the heterostructure. However we observe that, for the hBN/hBN interface, blisters are mobile in some samples but not in others. They can also be pinned at the SLG edge (see the right-hand edge of the dashed white line in Fig. 2b and Supplementary Fig. 2). This can result in samples where the SLG region is blister-free, but surrounded at the edges by blisters. Such blisters would not pose a limitation for large area heterostructure production, as this edge contamination does not affect the quality of the material in between, and can be removed by reactive ion etching^19^.

We do not see evidence of defects created by the CF while it moves across the heterostructure, as indicated by the lack of D peak in Raman spectra and by the consistently high, up to 1.8 × 10^6^ cm^2^ V^−1^ s^−1^, mobility at *T* = 9 K of the cleaned samples. Dissolving the PC film in chloroform post-cleaning would likely leave PC residuals on the top hBN surface. However, these would be isolated from the SLG by the top hBN, and therefore have no effect on the SLG.

**Fig. 3:**
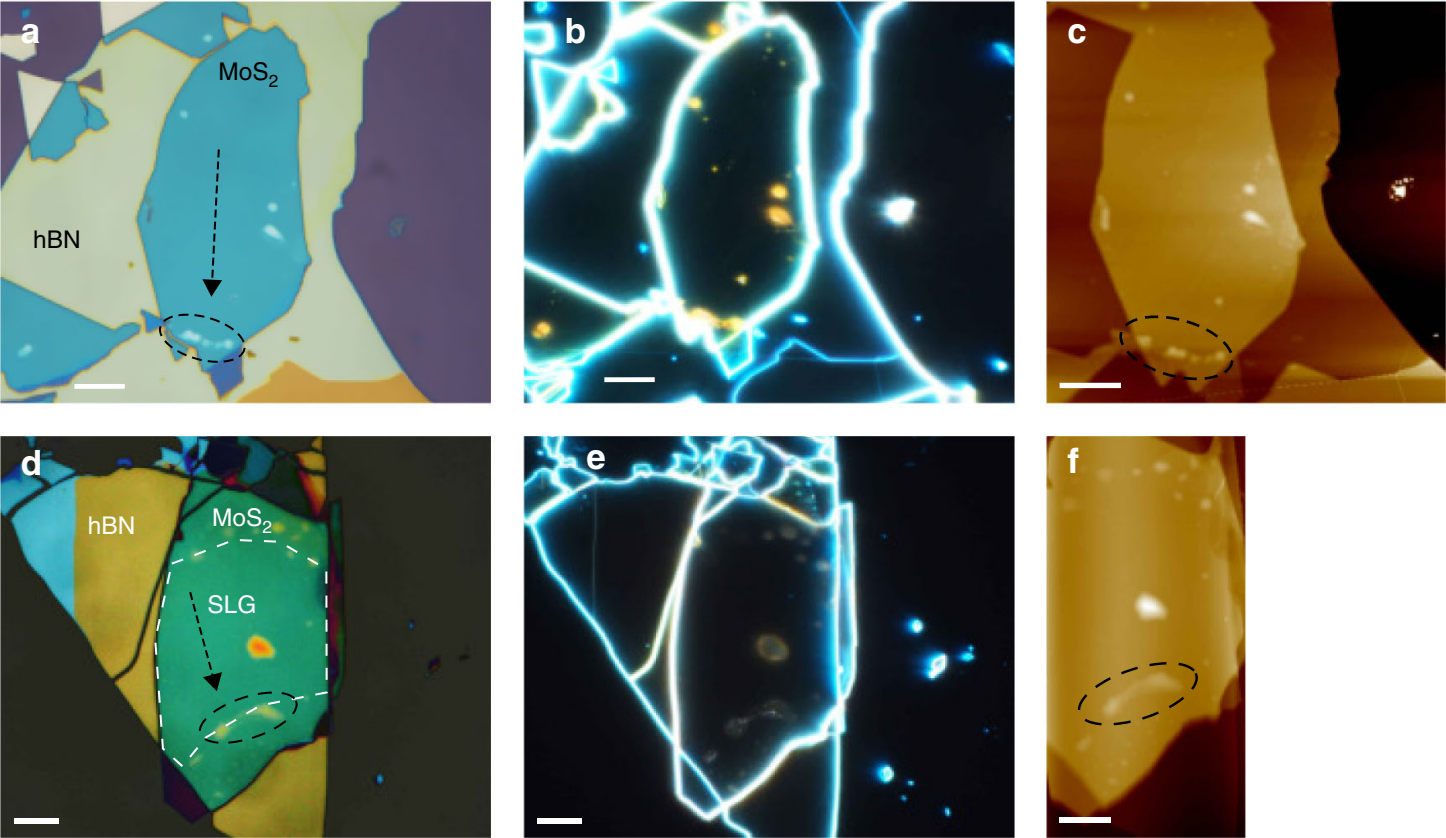
Blister manipulation in heterostructures containing MoS_2_. **a**–**c** Bright field, dark field, and AFM images of MoS_2_/hBN after cleaning. **d**–**f** Bright field, dark field, and AFM images of hBN/SLG/MoS_2_ after cleaning. In **a**, **c**, **d**, **f** the black dashed circles mark where the majority of blisters have been pushed. The arrows indicate the direction of the blister cleaning. The white dashed line in d shows the location of the SLG within the heterostructure. Scale bars 10 μm

Our cleaning method also works for heterostructures based on different materials, such as hBN/MoS_2_ and hBN/SLG/MoS_2_, as shown in Fig. 3, with blister manipulation and interface cleaning observed in these samples (see Supplementary Movies 4 and 5). Raman maps of these samples are reported in Supplementary Figs. 6 and 7.

**Fig. 4:**
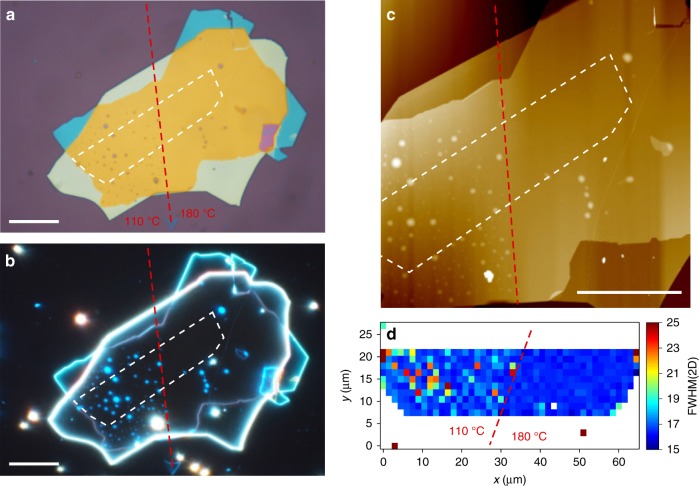
Effect of the temperature on blister cleaning. **a** Optical image of a sample partially cleaned at 110 and 180 °C. **b**, **c** Optical dark field and AFM images of the same sample. **d** FWHM(2D). The interface between the regions cleaned at 110 and 180 °C is marked by a dashed red line. The SLG location is marked by a while dashed line. Scale bars 20 μm

Ref. ^23^ reported that temperature plays a key role in the ability to exclude contaminants from heterostructure interfaces. Thus, we now consider the effectiveness of blister manipulation at 110 and 180 °C. In the cleaning step, we initiate the process at 110 °C, until the CF has passed half way across the sample, then we raise the temperature to 180 °C and advance the CF over the remaining portion of the heterostructure. Figure 4a–c are optical bright field and dark field images, and an AFM scan of the sample. In the portion of the heterostructure cleaned at 110 °C numerous blisters can be observed, while the portion cleaned at 180 °C is blister-free. This demonstrates the effect of temperature on the cleaning process, and highlights the difference in blister coverage between a cleaned and un-cleaned portion of sample. At 110 °C the mobility of the blisters is insufficient for them to be manipulated, while at 180 °C they are mobile and can be removed from the heterostructure.

### Analytical model

To understand the effect of temperature we consider a model based on quantized fracture mechanics^47^. In a stack formed by PDMS, PC, hBN, SLG, and hBN, laminated onto SiO_2_ (as in Fig. 1g), we can evaluate the elastic energy per unit length stored in the heterostructure around the zone of separation from the substrate (i.e., the curved region in Fig. 1g). This can be written as: ${\textstyle{{{\mathrm{d}}L} \over {{\mathrm{d}}s}}} = {\textstyle{1 \over 2}}{\textstyle{{\rm EI} \over {R^2}}}$, where *R* is the radius of curvature of this zone and EI is the heterostructure rigidity (i.e., the Young’s modulus multiplied by the moment of inertia of the cross-section of the stack, in N × m^2^). Considering the 5 materials in the stack (PDMS, PC, hBN, SLG and hBN), each with Young’s moduli *E*_*i*_ and thickness *h*_*i*_, we first derive the position of the elastic neutral axis (i.e., where the stresses are 0)^48^: *y*_0_ = ${\textstyle{{\mathop {\sum}\nolimits_{i = 1}^N {\kern 1pt} E_ih_iy_i} \over {\mathop {\sum}\nolimits_{i = 1}^N {\kern 1pt} E_ih_i}}}$, where *y*_*i*_ are the positions of the barycenters of each layers. We get^48^ EI = ${\textstyle{b \over {12}}}\mathop {\sum}\nolimits_{i = 1}^N \left[ {E_ih_i^3 + 12E_ih_i\left( {y_i - y_0} \right)^2} \right]$, where *b* is the width of the stack. For a homogeneous layer with Young’s modulus *E* and total thickness $h = \mathop {\sum}\nolimits_{i = 1}^N {\kern 1pt} h_i$, we have^49^ EI = $E{\textstyle{{bh^3} \over {12}}}$, where *I* [m^4^] is the momentum of inertia of the layer. Equating the last two expressions we get the homogenized Young’s modulus *E*_homog_ of the stack. During adhesion, the energy balance imposes^47^ ${\textstyle{{{\mathrm{d}}L} \over {{\mathrm{d}}s}}}$ = $2{{\Gamma }}b$, where Γ is the adhesion energy (in J/m^2^) between the stack and the substrate. The pressure generated at the interface is thus^47^ $p \cong {\textstyle{{{\Gamma }} \over R}} = 4\sqrt {{\textstyle{{3{{\Gamma }}^3} \over {Eh^3}}}}$. The pressure inside a circular-shaped blister of radius *a* needed for its propagation is^50^ $p_c \cong \sqrt {{\textstyle{{2\alpha \gamma _{i,j}E} \over {\pi (a + q{\mathrm{/}}2)}}}}$, where *γ*_*i*,*j*_ is the adhesion energy between two layers *i*, *j* (i.e., hBN and SLG) forming the blister, *α* is a non-dimensional shape factor close to unity^47^, and *q* is the minimum value of blister advancement. The condition for blister cleaning is *p* > *p*_c_. Noting that the adhesion energies are *T*-dependent and present maximal values at a given *T* (e.g., SLG’s adhesion to SiO_2_ is maximum at ~250 °C^51^), we get Γ = Γ^(max)^*f*(*T*) and $\gamma _{i,j} = \gamma _{i,j}^{({\mathrm{max}})}g\left( {{T}} \right)$, where 0 < *f*(*T*), *g*(*T*) ≤ 1. Similarly, *E*_*i*_(*T*) are *T*-dependent, thus *E*(*T*) = *E*^(max)^e(*T*), where 0 < e(*T*) ≤ 1. Accordingly, for blister cleaning the following condition must be satisfied:

1 $$C({{T}}) = \frac{{f({{T}})^3}}{{{\mathrm e}({{T}})^2g({{T}})}} > \frac{{\alpha E^{({\mathrm{max}})2}h^3\gamma _{i,j}^{({\mathrm{max}})}}}{{24\pi (a + q{\mathrm{/}}2){{\Gamma }}^{({\mathrm{max}})3}}} = A$$

where we introduced the dimensionless cleaning thermal driving force *C*(*T*) and the blister resistance *A*. By increasing *T* we can simultaneously increase *C*(*T*) and decrease *A*, e.g., by reducing *E*^(max)^ imposing a glass transition of a polymer layer. Thus, in our case, well above the PC *T*_g_, *E*_PC_ becomes negligible. For perfect cleaning *a* = 0 and *A* is maximal. Considering *f*(*T*) ≅ *g*(*T*) (same *T* dependence of *γ*_*i*,*j*_ and Γ) and e(*T*) ≅ 1 (nearly *T*-independent homogenized *E*), the blister cleaning requires *T* in the range *T*_0_ − Δ*T*_−_ ≤ *T* ≤ *T*_0_ − Δ*T*_+_, where *T*_0_ is the *T* at which surface energies are maximal, i.e., *f*(*T*_0_) = *g*(*T*_0_) = 1 (note that Δ*T*_−_ = Δ*T*_+_ if a symmetric function is assumed). In this case, the condition for blister cleaning becomes *C*(*T*) ≅ *g*(*T*)^2^ > *A*. Considering the *T* dependence of the adhesion energy for SLG on SiO_2_^51^, we can assume *T*_0_ ≅ 250 °C. Noting that for PC, *T*_g_ ≅ 150 °C, we expect a 150–250 °C range of minimal *T* for blister cleaning, in good agreement with our observation of no blister cleaning below 150 °C and good cleaning at 180 °C.

Our model explains why the condition for blister cleaning (i.e., the temperature at which blisters become mobile) depends on the materials forming the heterostructure. Whereas a temperature of 180° works well for all hBN/SLG/hBN heterostructures, this is not always true at the hBN/hBN interface. The temperature needed for blister cleaning depends on *E*_*i*_, *h*_*i*_, and *γ*_*ij*_. hBN interfaces requires/hBN interfaces, the difference in *γ* (assuming all the other parameters identical) would require a different temperature. Ref. ^52^ gives *γ*_*ij*_ ≈ 84.7 meV/atom for SLG/SLG, ≈85.9 meV/atom for hBN/hBN and ≈58.3 meV/atom for SLG/hBN. Therefore the increment in γ_*ij*_ at hBN interfaces requires/hBN interfaces requires larger temperatures, explaining why blister manipulation is achieved when graphene is sandwiched between two hBN, but not always at the hBN/hBN interfaces.

### Raman spectroscopy

**Fig. 5:**
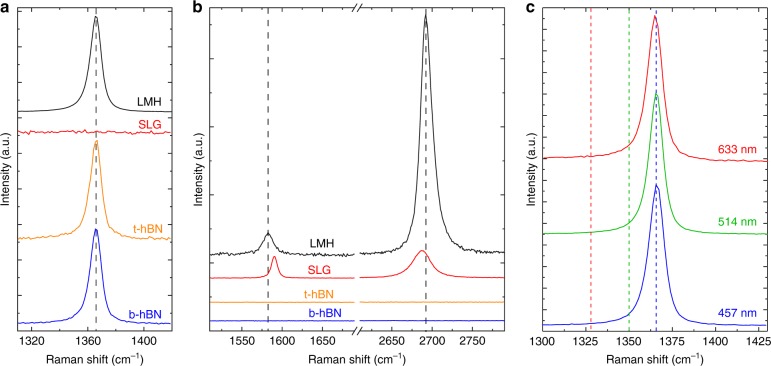
Raman spectra before and after heterostructure assembly. The bottom hBN flake (b-hBN) is shown in blue, the top hBN flake (t-hBN) in orange, the SLG flake in red, and the assembled heterostructure in black. **a** hBN E_2g_ spectral region. The measured spectra are normalized to the height of the E_2g_ peak. **b** G and 2D peak spectral region. The spectra containing SLG peaks are normalized to the height of the G peak. The spectra associated with t-hBN and b-hBN have the same scaling as in a. The E_2g_, G and 2D peaks after encapsulation are marked by dashed black lines. Spectra in **a** and **b** are acquired at 514 nm. **c** Raman spectra measured at 457, 514, and 633 nm. The expected Pos(D) at 457, 514, and 633 nm are shown by dashed lines in blue, green and red, respectively. The spectra are normalized to the height of the 2D peak

The quality of the flakes is monitored both before and after assembly by Raman spectroscopy. Figure 5a–c plots the spectra of a typical sample, with 92 and 176 nm thickness top and bottom hBN flakes. Figure 5a shows that the E_2g_ peak for both the bottom and top hBN are at 1366 cm^−1^, with full-width half maximum (FWHM) ≈ 9.2 and 8.6 cm^−1^, respectively, as expected for bulk hBN^40,43,53^. The SLG G and 2D peaks before transfer are plotted in Fig. 5b. The 2D peak can be fit with a single Lorentzian, with FWHM(2D) ≈ 26 cm^−1^, and position Pos(2D) ≈ 2687 cm^−1^, as expected for SLG^41,42^. No D peak can be seen, indicating negligible defects^41,42,54^. The position of the G peak, Pos(G) ≈ 1590 cm^−1^, FWHM(G) ≈ 8 cm^−1^, and the intensity and areas ratio of the 2D and G peaks, I(2D)/I(G) ≈ 1.3, A(2D)/A(G) ≈ 3.9 indicate that the sample is doped with *E*_F_ ≳ 300 meV^55,56^. The spectrum of the assembled heterostructure is shown in black in Fig. 5b. The hBN E_2g_ peak is now a combination of those of both top and bottom hBN. This yields a single peak with Pos(E_2g_) ≈ 1366 cm^−1^ and FWHM(E_2g_) ≈ 9.3, as expected considering both flakes are bulk^40,43,53^. For the encapsulated SLG we have Pos(2D) ≈ 2693 cm^−1^, Pos(G) ≈ 1583 cm^−1^, FWHM(G) ≈ 15 cm^−1^, I(2D)/I(G) ≈ 11.4 cm^−1^ and A(2D)/A(G) ≈ 12.9 cm^−1^, indicating $E_{\mathrm{F}} \ll 100$ meV^55,56^. FWHM(2D) decreases to ≈17 cm^−1^ after encapsulation, indicating a reduction in the nanometer-scale strain variations within the sample^57–60^. We note that the E_2g_ peak of hBN may overlap the D peak. This is a general issue in hBN-encapsulated samples. However, the D peak shifts with excitation energy by^42^ ≈50 cm^−1^/eV due to a combination of its double resonance activation^40,42^ and a Kohn Anomaly at the *K* point of the Brillouin Zone^61^, while the E_2g_ of hBN does not, since hBN has a band gap and no Kohn anomalies nor double resonances are present^40,61^. Figure 5c compares the Raman spectra at 457, 514, and 633 nm. No D peak is seen even at 633 nm, where it should be well clear of the E_2g_ of hBN, thus ensuring no extra defects are introduced in the SLG by the transfer and cleaning processes.

Following encapsulation and blister removal, we process our heterostructures into Hall-bars for 4-terminal transport measurements (see Methods for details). We fabricate Hall bars with *W* up to = 24 μm, exploiting the entire heterostructure dimensions. For comparison, for samples containing blisters *W* ~ 1–3 μm is typical^20,22,30^.

**Fig. 6:**
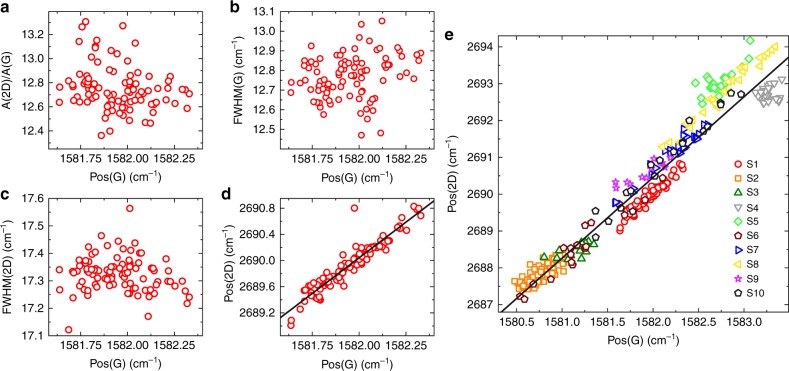
Raman fitting parameters of hBN/SLG/hBN Hall bars. **a**–**d** Pos(2D), A(2D)/A(G), FWHM(2D), FWHM(G) as a function of Pos(G) measured across a 20 μm × 20 μm region of a Hall bar (optical image shown in the inset of Fig. 7). **e** Pos(2D) vs. Pos(G) for 10 samples (S1-S10). S1 corresponds to the measurements in **a**–**d**. Solid black lines represent linear fits to the data

**Fig. 7:**
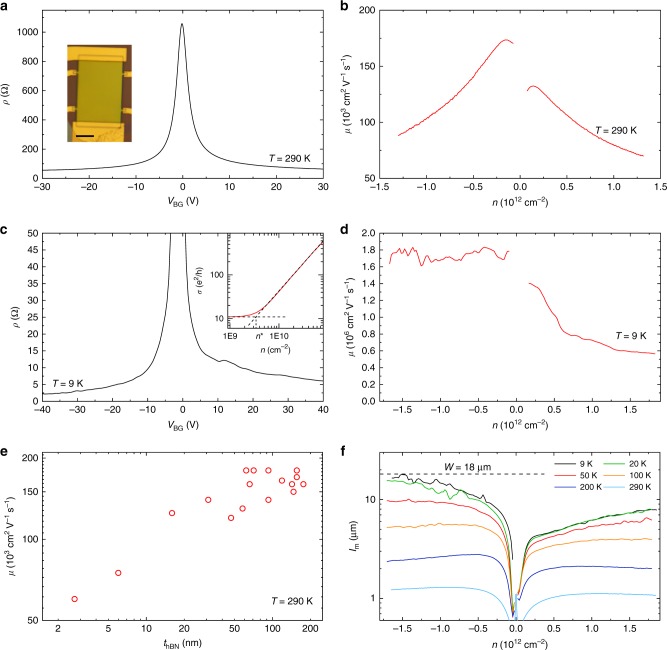
Four terminal transport measurements. **a** Resistivity as a function of back gate voltage at room temperature. Inset: optical image of the measured Hall bar with *W* = 24 μm. Scale bar 10 μm. **b** Mobility *μ* = *σ*/(*n*e) at room temperature. *n* is extracted from a Hall measurement with a *B* = 0.5T out of plane magnetic field. **c** Resistivity of the sample at 9 K. Inset: conductivity as a function of carrier density close to the CNP, showing the extraction of n^*^. The dashed lines represent extrapolated fits to the linear and sloped portions of the conductivity. **d** Extracted mobility as a function of charge carrier density at 9 K. **e** Room temperature mobility of 17 encapsulated SLG samples as a function of the bottom hBN thickness. The mobility is the peak value of the density dependent mobility, i.e., the mobility close to the CNP. **f** Mean free path as a function of carrier density for different temperatures for a sample with width *W* = 18 *μ*m

We then perform Raman mapping after device fabrication. The data in Fig. 6a–d are taken from a ≈20 μm × 20 μm map on the Hall bar in the inset in Fig. 7a. Pos(G) is sensitive to both doping^56^ and strain^62^, meaning that local variations in these quantities manifest as a spread in the G peak position, i.e., ΔPos(G). From Figures 6a–d ΔPos(G) ≈ 0.6 cm^−1^. Figure 6a, b plot A(2D)/A(G) and FWHM(G) as a function of Pos(G), showing no correlation. This indicates that the contribution to ΔPos(G) due to doping is negligible^56,60,61,^ and that the trend in Fig. 6d is due to strain $\left( \epsilon \right)$. Figure 6d plots Pos(2D) as a function of Pos(G). A linear correlation can be seen with slope ΔPos(2D)/ΔPos(G) ≈ 2.18. A similar trend was reported in ref. ^63^, with a slope ≈2.2.

The rate of change of Pos(2D) and Pos(G) with strain is determined by the Grüneisen parameters^62^, which relate the relative change in the peak positions in response to strain, i.e., [*ω* − *ω*_0_]/[2$\epsilon$*ω*_0_], where *ω* is the frequency of the Raman peak at finite strain and *ω*_0_ the frequency at zero strain^62^. For biaxial strain the Grüneisen parameters for G and 2D peak are *γ*_G_ ≈ 1.8 and *γ*_2D_ ≈ 2.6, resulting in ΔPos(2D)/ΔPos(G) ≈ 2.5^62,64,65^. In the case of uniaxial strain *γ*_G_ ≈ 1.8^62^, however extraction of *γ*_2D_ is not straightforward, as uniaxial strain shifts the relative position of the SLG Dirac cones^62,64^, which in turn effects the 2D peak as it is activated by an intervalley scattering process^42,62^. Ref. ^62^ determined an upper bound *γ*_2D_ ≈ 3.55 and theoretically calculated *γ*_2D_ ≈ 2.7, consistent with experimentally reported ΔPos(2D)/ΔPos(G) ≈ 2–3^62,63,66^. Biaxial strain can be differentiated from uniaxial from the absence of G and 2D peak splitting with increasing strain^42^, however at low (≲0.5%) strain the splitting cannot be resolved^62,66^. Due to these factors the presence (or coexistence) of biaxial strain cannot be ruled out in our samples. For uniaxial(biaxial) strain, Pos(G) shifts^62,64,66^ by ΔPos(G)/$\Delta \epsilon$ ≈ 23 (60) cm^−1^%^−1^. For intrinsic SLG ($E_{\mathrm{F}} \ll 100$ meV), the unstrained value of Pos(G)is ≈ 1581.5 cm^−1^ for 514 nm excitation^41^. For the sample in Fig. 6d, ΔPos(G) ≈ 0.6 cm^−1^ equates to $\Delta \epsilon$ ≲ 0.026%. The average Pos(G) ≈ 1582 cm^−1^ indicates an average strain $\epsilon$ ≈ 0.025%. Figure 6e plots Pos(2D) as a function of Pos(G) for 10 samples (S1–S10) encapsulated using *t*_hBN_ > 10 nm. It shows a linear trend, with a slope ≈2.19. ΔPos(G) ranges from ≈0.5 to 2 cm^−1^, indicating differences in $\Delta \epsilon$ up to a factor ≈4. The average Pos(G) for each sample varies from 1580.8 to 1583.5 cm^−1^, indicating different strains. For example, since Pos(G) ≈ 1581.5 cm^−1^ for zero strain^41,61^, sample S2 has an average tensile $\epsilon$ ≈ 0.03% while sample S4 has an average compressive strain $\epsilon$ ≈ 0.09%. The maximum absolute strain is $\epsilon$ ≈ 0.1% in sample S4.

Ref. ^57^ reported a Raman map of SLG encapsulated in hBN containing blisters. Pos(G) and Pos(2D) varied by ≳5 cm^−1^ and ≳15 cm^−1^ across ~200 μm^2^. $\Delta \epsilon$ in ref. ^22^ was ≈0.2–0.3%, around one order of magnitude larger than in our samples. Ref. ^57^ detected FWHM(2D) > 20 cm^−1^ over blisters, as compared to blister-free regions where they found FWHM(2D) < 20 cm^−1^. A similar behavior can be observed in Fig. 4d, where the blisters in the portion of the sample cleaned at 110 °C appear as spots with increased FWHM(2D) in the Raman map, while FWHM(2D) in the portion cleaned at 180 °C is homogeneous (spread < 1 cm^−1^) and narrow (<17 cm^−1^).

### Transport

Figure 7 shows 4 terminal measurements of hBN/SLG/hBN Hall bars. Figure 7a plots the resistivity (*ρ*) as a function of back gate voltage *V*_BG_. Carrier density (*n*) as a function of *V*_BG_ is extracted from a measurement of the Hall voltage with a B = 0.5 T out of plane magnetic field. From a linear fit of the dependence of *n* vs. *V*_BG_ we get a gate capacitance of *C*_ox_ = 7 × 10^−5^ Fm^−2^. This is in agreement with that calculated assuming a parallel plate capacitor with a bottom hBN flake in series with 285 nm SiO_2_. The bottom hBN thickness is 156 nm extracted from AFM. We take its dielectric constant $\epsilon _{\mathrm{r}}$ = 3, considering that values between 2–4 are usually reported^27^. This gives *C*_ox_ = 7.1 × 10^−5^ Fm^−2^. We note that *C*_ox_ is orders of magnitude smaller than the quantum capacitance of SLG^67^, which is therefore neglected in the calculations. The sample is highly intrinsic, with a charge neutrality point (CNP) at *V*_BG_ of *V*_0_ = −0.2V, corresponding to a residual *n*_0_ = (*C*_ox_/e)*V*_0_ = 9 × 10^9^ cm^−2^.

The carrier density dependent mobility is extracted assuming a Drude model of conductivity *μ* = *σ*/(*n*e), as shown in Fig. 7b. The peak mobility close to the CNP is ≈180,000 cm^2^ V^−1^ s^−1^, decreasing at higher densities. Of 13 Hall bars with *W* ranging from 3 up to 24 μm, all exhibit peak room temperature mobilities >100,000 cm^2^ V^−1^ s^−1^. The conductivity (*σ*) of SLG is commonly fit using *σ*^−1^ = (*n*e*μ*_L_ + *σ*_0_)^−1^ + *ρ*_s_, where *μ*_L_ represents the contribution from long-range scattering, and *ρ*_s_ the density independent contribution from short-range scattering^18,22,68^. *ρ*_s_ results in a sublinear dependence of *σ* with *n* and therefore decreasing *μ* with increasing *n*. Fitting the data of Fig. 7a yields *μ*_L_ = 217,000 cm^2^ V^−1^ s^−1^ and *ρ*_s_ = 33 Ω. For encapsulated samples at room temperature, the dominant contribution to *ρ*_s_ has been attributed to electron-phonon scattering *ρ*_e-ph_^19^, which sets an upper bound on the achievable *μ* = 1/(*n*e*ρ*_e-ph_). At 290 K the theoretically predicted *ρ*_e-ph_ ~ 32 Ω^69,70^ is consistent with our extracted value *ρ*_s_ = 33 Ω. For *n* = 9 × 10^12^ cm^−2^ we measure *μ* ~ 19,000 cm^2^ V^−1^ s^−1^ (see Supplementary Fig. 4), close to the phonon limit ~21 000 cm^2^ V^−1^ s^−1^ calculated for this density^69^.

The resistivity of the same sample at 9 K (corresponding to the base temperature for our measurement system) is plotted in Fig. 7c. *μ* as a function on *n* at this temperature is shown in Fig. 7d, with a peak value ~1.8 × 10^6^ cm^2^ V^−1^ s^−1^. We note that for p-doping, *μ* remains above 1.5 × 10^6^ cm^2^ V^−1^ s^−1^ even at *n* > 1 × 10^12^ cm^−2^, in close agreement with ballistic measurements on SLG encapsulated in hBN at similar *n*^19–21^. Assuming diffusive transport, i.e., *l*_*m*_ < *W*^20^, we can write $l_m = \left( {h{\mathrm{/}}2{\mathrm e}^2} \right)\sigma \left( {1{\mathrm{/}}\sqrt {n\pi } } \right)$^71^, meaning *l*_*m*_ ∝ *σ* for a given *n*. As the lateral dimensions of the sample constrain *l*_*m*_ ≲ *W*^20,30^, *W* sets an upper bound on the achievable *σ*, and therefore *μ*, for a particular value of *n*. Achieving *μ* = 1.7 × 10^6^ cm^2^ V^−1^ s^−1^ at *n* = 1.5 × 10^12^ cm^−2^ can therefore be seen as a direct result of *W* > 20 *μ*m.

The CNP FWHM, *δV*, as a function of carrier density, *δn* = (*C*_ox_/e)*δV*, places an upper bound on the disorder induced charge inhomogeneity, n^*^^22,72,73^. From the measurements in Fig. 7c *δn* = 10^10^ cm^−2^, almost an order of magnitude lower than typical reports for SLG on hBN^18,30^. A more precise n^*^ can be extracted by fitting the linear and plateau regions of *σ* at the CNP^72,74^ (inset in Fig. 7c), giving n^*^ = 3.5 × 10^9^ cm^−2^. n^*^ provides a measure of the spatial inhomogeneity of the carrier density close to the CNP^75^, which arises due to disorder (e.g., local variations in strain^76^, or chemical doping^77^). Lower n^*^ are indicative of less disordered, more homogeneous samples. Our n^*^ = 3.5 × 10^9^ cm^−2^ is approximately three times lower than typical n^*^ > 1 × 10^10^ cm^−2^ for SLG encapsulated in hBN^22,30^.

Figure 7e shows the mobility of seventeen different samples at room temperature as a function of the bottom hBN thickness. A clear increase in mobility with *t*_hBN_ is seen. The maximum values of mobility are achieved for *t*_hBN_ ≥ 15 nm, above which the mobility plateaus out. This can be attributed to screening of the roughness and charged impurities of the underlying SiO_2_^78^. Indeed, the roughness of hBN on SiO_2_ shows an equivalent trend, with atomic flatness achieved only for *t*_hBN_ ≳ 15 nm^18^. *l*_*m*_ extracted from a Hall bar with *W* = 18 μm is plotted in Fig. 7f between 9 and 290 K. The sample width is marked by a dashed line, showing that *l*_*m*_ < *W* for all carrier densities and temperatures, indicating transport remains diffusive^20^. The values of *l*_*m*_ are in close agreement with ref. ^19^ where a 15 × 15 μm square sample free of blisters was measured. Transport properties of encapsulated bilayer graphene are reported in Supplementary Fig. 3.

### Cleaning of polymer-contaminated samples

Our method also works for heterostructures where the SLG surface is exposed to polymers and solvent before encapsulation, which is a common occurrence when the SLG undergoes lithographic processing^23^ or wet and (or) polymer-assisted transfer used to process large-area SLG films^22,36,37^. To demonstrate this, we spin coat PMMA onto exfoliated SLG on SiO_2_ + Si. PMMA is then removed by rinsing in Acetone/IPA. SLG is then encapsulated following the same procedure as in Fig. 1. The only modification is that cleaning (Fig. 1g) is performed at 250 °C, as we find the blisters remain immobile at 180 °C in these samples. This need for higher temperature cleaning could be attributed to the increased amount of contaminants trapped at the interfaces in these samples. This is in agreement with the analytical model, which predicts optimal cleaning at *T*_0_ ~ 250 °C.

**Fig. 8:**
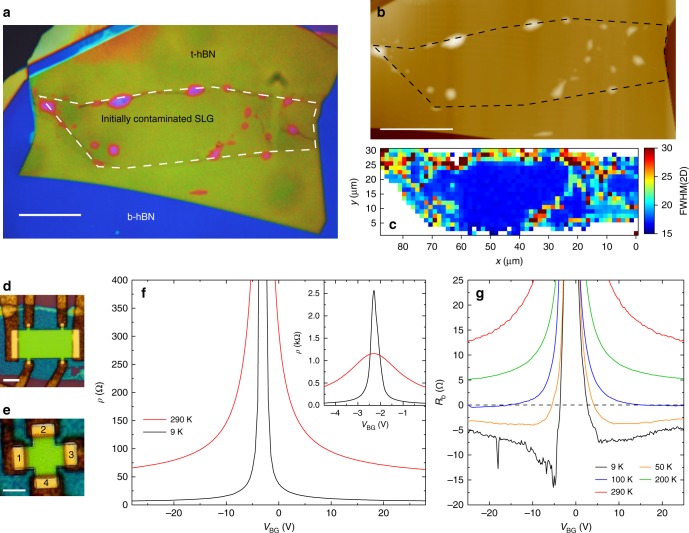
Characterization of heterostructures produced using SLG exposed to both polymer residuals and solvents. **a** False color optical image. SLG is indicated by the white dashed line. Blisters have been pushed to the SLG edges. Scale bar 20 μm. **b** AFM scan of the sample. The black dashed line shows the SLG. **c** Spatial map of FWHM(2D) of the sample, taken at an excitation wavelength of 514 nm. **d** Hall bar processed from the sample. Scale bar 5 μm. **e** Hall cross with arm width 2 μm from the same heterostructure. Scale bar 2 μm. The contacts are labeled 1–4. **f** Resistivity at 9 and 290 K. Inset: Resistivity at 9 and 290 K plotted close to CNP. **g** Bend resistance measurements of Hall cross in **b** as a function of temperature

Figure 8a show optical image of the cleaned heterostructure, with the SLG indicated by a white dashed line. Figure 8b is an AFM scan, with the SLG marked by a dashed black line, from which it can be seen that the blisters have been pushed to the SLG edge. A few blisters remain within the SLG, pinned by folds and cracks. A FWHM(2D) map across the sample is shown in Fig. 8c. The blister-free region exhibits homogeneous (spread < 1 cm^−1^) and narrow (~17 cm^−1^) FWHM(2D), consistent with uncontaminated SLG (see Fig. 4d).

We measure the mobility of our initially polymer contaminated SLG samples by processing them into 4-terminal geometries. Figure 8d, e respectively show a Hall bar and Hall cross processed from the sample in Fig. 8a. Figure 8f plots the resistivity extracted from the Hall bar at 290 and 9 K. We get *μ* ~ 150,000 cm^2^ V^−1^ s^−1^ at 290 K and 1.3 × 10^6^ cm^2^ V^−1^ s^−1^ at 9 K, and n^*^ ~ 5.5 × 10^9^ cm^−2^. For comparison refs. ^17,18^ used SLG on hBN (un-encapsulated) where the SLG surface was also exposed to polymers and solvents, and reported *μ* ~ 50,000–100,000 cm^−2^ V^−1^ s^−1^ at *T* < 10 K. Ref. ^30^ used encapsulated SLG in hBN, where the SLG was exposed to solvent and polymer residue before encapsulation, achieving *μ* ~ 150,000 cm^−2^ V^−1^ s^−1^ at *T* < 10 K. We achieve mobilities an order of magnitude higher, demonstrating the effectiveness of our technique.

In order to further confirm the cleanliness of the interfaces in the heterostructure containing initially polymer contaminated SLG we also investigate ballistic transport. To the best of our knowledge, micrometer scale ballistic transport in SLG was only reported in the highest quality SLG encapsulated in hBN samples^19–21^, where the interfaces are clean^19,31^, and $\mu \gg 100,000$ cm^2^ V^−1^ s^−1^. Ballistic transport is commonly probed using bend resistance measurements^19–21,79^, where current is applied around a bend in a sample and the corresponding voltage developed measured. We perform these measurements on the Hall cross shown in Fig. 8e, with arm width *H* = 2 μm. A current is passed from contact 1 to 2 (*I*_1,2_), while measuring the voltage drop between contacts 4 and 3 (*V*_4,3_). The bend resistance is defined as *R*_B_ = *V*_4,3_/*I*_1,2_^20^. For diffusive transport, where $l_{{m}} \ll H$, carriers travel diffusively around the bend, and *R*_B_ is positive and determined by the van-der-Pauw formula^20^ *R*_B_ = *ρπ*/ln 2. However if *l*_*m*_ exceeds *H*, carriers injected at contact 1 travel ballistically to contact 3, resulting in negative *R*_B_^20,79^. A negative *R*_B_ therefore imposes *l*_*m*_ > *H*, from which a lower bound on the mobility can be calculated from $\mu = (2{\mathrm e}{\mathrm{/}}h)l_{{m}}\sqrt {\pi {\mathrm{/}}n}$ where *l*_*m*_ > *H*^19,21^. Figure 8g plots *R*_B_ as a function of temperature. At 9 K and *n* = 1.1 × 10^12^ cm^−2^ we estimate *μ* > 520,000 cm^2^ V^−1^ s^−1^. At 290 K the mobility extracted diffusively from the cross is *μ* ~ 150,000 cm^2^ V^−1^ s^−1^. These measurements are consistent with those on the highest mobility encapsulated SLG in literature where room temperature mobilities ~150,000 cm^2^ V^−1^ s^−1^ are achieved^19,20,30^, demonstrating that exposure of the SLG surface to polymers or solvents before encapsulation poses no limitations once the appropriate cleaning procedure is used.

## Discussion

We developed a transfer method that allows blisters to be mechanically manipulated, and removed from layered material heterostructures. This enabled us to achieve blister-free regions of SLG encapsulated in hBN limited only by the size of the exfoliated flakes. We achieved mobilities up to ≈180,000 cm^2^ V^−1^ s^−1^ at room temperature, and ≈1.8 × 10^6^ cm^2^ V^−1^ s^−1^ at 9 K. Our method can be used to clean encapsulated samples assembled with polymer contaminated SLG, and these show equivalent mobilities, up to ≈150,000 cm^2^ V^−1^ s^−1^ at room temperature, indicating that the polymer and solvent residuals can be removed from the SLG/hBN interface. Our method provides consistent results, as shown in Supplementary Table 1, which summarized transport and Raman measurements of 18 encapsulated SLG Hall bars. Finally, our approach is general and can be used for other heterostructures.

## Methods

### Layered material synthesis and micro-mechanical cleavage

hBN single crystals are grown under high pressure and high temperature, as detailed in ref. ^80^. The graphite is first cleaved using adhesive tape. The Si + SiO_2_ substrate is then exposed to an oxygen plasma (100 W, 360 s). The surface of the tape is brought into contact with the SiO_2_ substrate, which is then placed on a hot plate at 100 °C for 2 min, before the tape is removed. Heating the substrate allows us to achieve large (>100 μm) SLG flakes, whereas flakes produced without heating are typically <50 μm in size, in agreement with findings of ref. ^50^. For the exfoliation of hBN, no plasma treatment of the SiO_2_ surface is used, as we find this has no effect on the flakes’ lateral size. Polymer-contaminated samples are produced by first exfoliating SLG and subsequently depositing PMMA (8% in Anisole, 495 K molecular weight) via spin coating at 4000 rpm for 60 s. PMMA is then removed by acetone and isopropyl alcohol.

### Stamp preparation

A PC film is prepared by drop casting a solution in chloroform (5% weight) onto a glass slide. A second slide is then used to sandwich and spread the solution between the two slides. The slides are immediately slid apart, and left to allow the chloroform to evaporate. After drying, the resultant film is picked up and mounted on a PDMS block (a few mm thick) to complete the stamp. A detailed description of the PC film preparation is reported in Supplementary Fig. 5.

### Device fabrication

The heterostructure is first dry etched, defining the geometry, as well as exposing the SLG edge. Depositing metal onto the exposed edges results in ohmic contacts between the SLG and metal^19^. We first deposit an Al mask using e-beam lithography, metal evaporation and lift-off. We then use a reactive ion etcher (RIE), with a forward radio frequency (RF) power of 20 W and a ≈20 sccm flow of CF_4_. The etch rate is ≈0.2 nm/s, with the total etch time set depending on the heterostructure thickness. After wet-etching to remove the Al mask, metal contacts are patterned by e-beam lithography followed by either e-beam evaporation and lift-off of 5/150 nm Cr/Au^19^, or DC sputtering and lift-off of 5/150 nm of Cr/Cu. We note that our contact success rate >90% is not affected by the thickness of the bottom hBN, which in some cases exceeds the thickness of the metal film. This is due to the anisotropic etching of the hBN when exposed to plasma, which consistently results in edges with a slope of 45–60°^19^. Upon evaporation or sputtering, the metal conformally coats both hBN surface and edge, resulting in a good contact with SLG. Using a hard (Al) mask increases contact yield, and lowers contact resistance, compared to conventional polymer etch masks.

### Characterization

Raman measurements are performed using a Renishaw inVia microspectrometer equipped with 457, 514, and 633 nm excitation wavelengths. AFM images are acquired using a Bruker Dimension Icon, operated in PeakForce mode.

### Transport measurements

Transport measurements are performed using a dual lock-in amplifier (Stanford Research Systems SR810 and SR860), combined with a low noise voltage pre-amplifier (Stanford Research Systems SR860) in a Lakeshore cryogenic probe station at ~3 × 10^−8^ Torr. A bias current of 100 nA and a lock-in frequency ~13 Hz are used at all temperatures.

## Electronic supplementary material

Supplementary Information

Description of Additional Supplementary Files

Supplementary Movie 1

Supplementary Movie 2

Supplementary Movie 3

Supplementary Movie 4

Supplementary Movie 5

## Data Availability

Data supporting the findings of this manuscript are available from the corresponding author upon reasonable request.
