# Supplementary material for: Cleaning interfaces in layered materials heterostructures
Source: Nat Commun. 2018 Dec 19;9:5387. doi: 10.1038/s41467-018-07558-3 (PMC6300598; doi:10.1038/s41467-018-07558-3)
Supplement: Supplementary file 1 — Supplementary Information [file 41467_2018_7558_MOESM1_ESM.pdf]

# Supplementary Information

## Cleaning Interfaces in Layered Materials Heterostructures

D. G. Purdie[1], N. M. Pugno[2,3,4], T. Taniguchi[5], K. Watanabe[5], A. C. Ferrari[1], A. Lombardo[1*]

[1] *Cambridge Graphene Centre, University of Cambridge, 9 JJ Thomson Avenue, Cambridge CB3 0FA, UK*
[2] *Laboratory of Bio-inspired and Graphene Nanomechanics, Department of Civil, Environmental and Mechanical Engineering, University of Trento, via Mesiano 77, I-38123 Trento, Italy*
[3] *School of Engineering and Materials Science, Queen Mary University of London, Mile End Road, London, E1 4NS, UK*
[4] *Ket-lab, E. Amaldi Foundation, Italian Space Agency, Via del Politecnico, Rome 00133, Italy*
[5] *National Institute for Materials Science, 1-1 Namiki, Tsukuba 305-0044, Japan*

*corresponding author: al515@cam.ac.uk

## Supplementary Figures

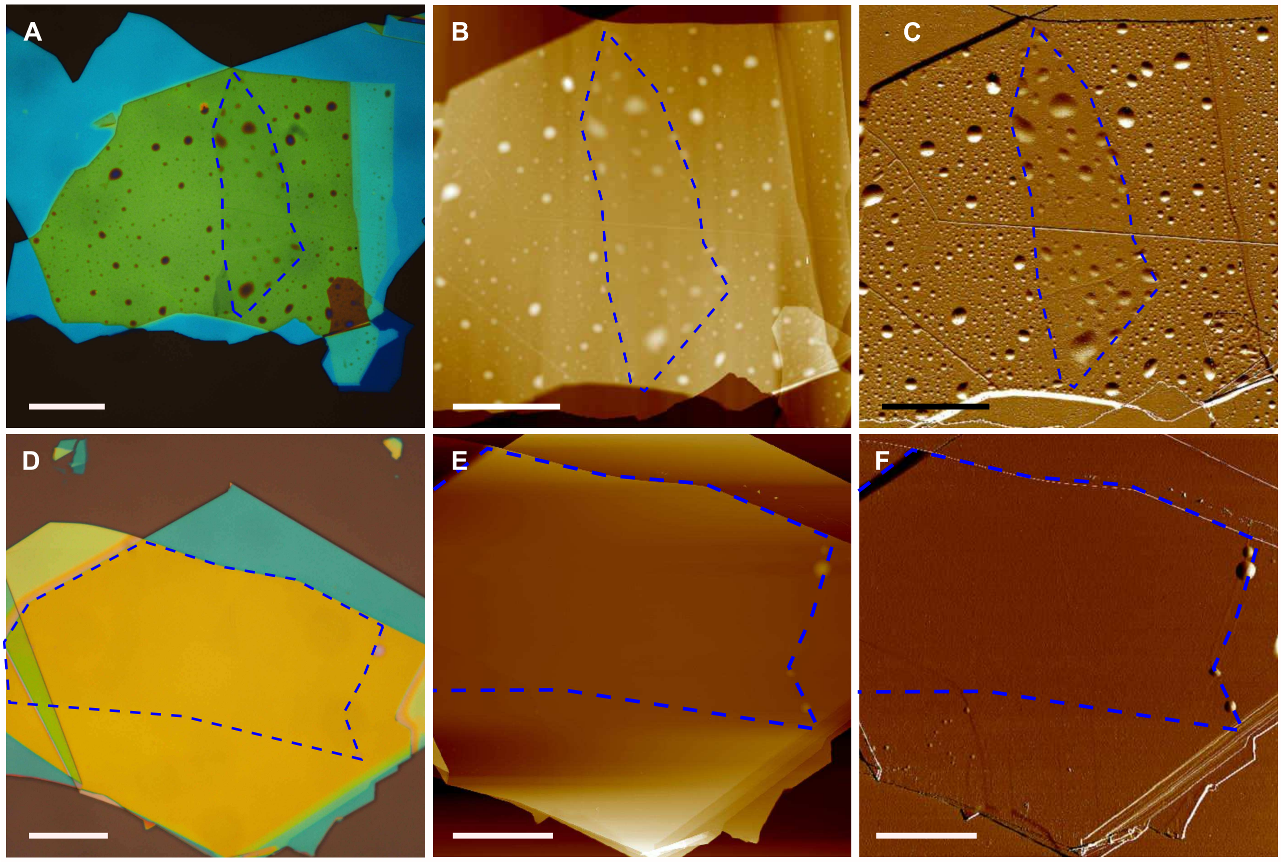


**Supplementary Figure** 1: Comparison of heterostructures with and without blister cleaning. a)-c) Optical false color, AFM topography, and AFM topography error images of a sample encapsulated without blister cleaning. d)-f) Optical false color, AFM topography, and AFM topography error images of a sample with blister cleaning (d and e are reproduced from Figure 2 in the main text). The blue dashed line represents the location of the SLG in the heterostructures. The reduction in blisters can clearly be observed in the error signal images (c and f).

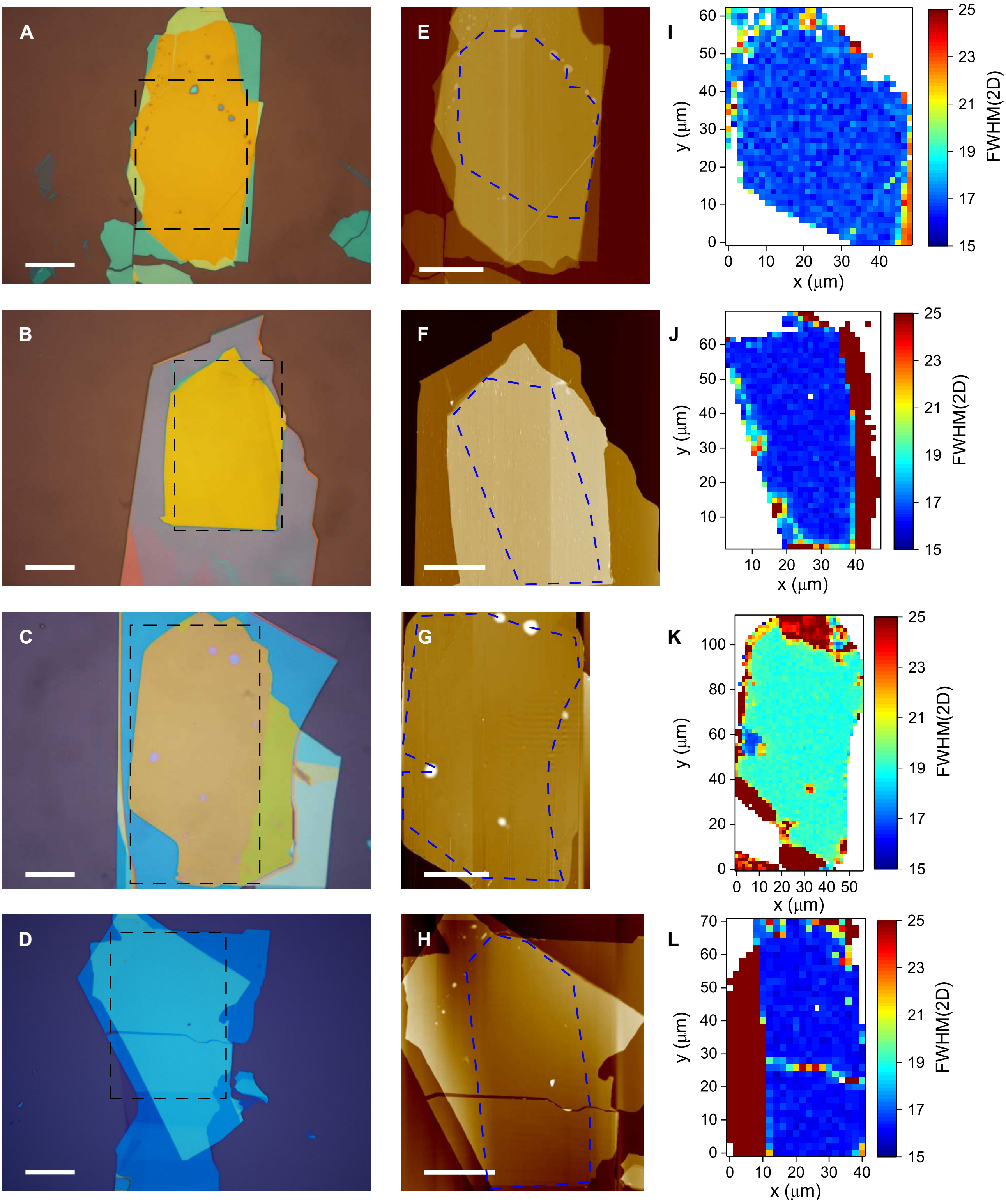


**Supplementary Figure** 2: Optical images, AFM, and Raman Maps of different hBN/SLG/hBN heterostructures cleaned of blisters. a)-d): Optical images of five different samples consisting of SLG encapsulated in hBN, prepared using our blister cleaning method. The corresponding AFM scans and Raman maps of FWHM(2D) are shown in e)-h): and i)-l): respectively. The location of the Raman maps is marked by the dashed black rectangle in a)-d). The location of the SLG within the samples is marked by the dashed blue line in e-h

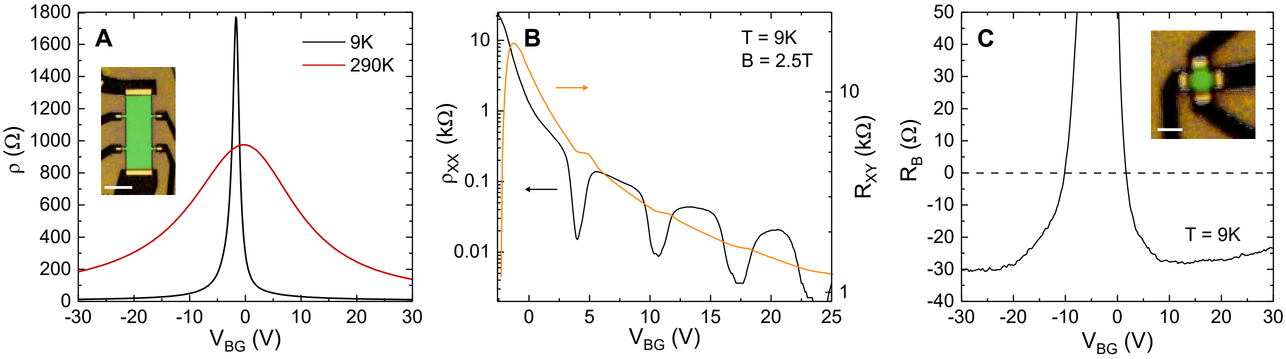


**Supplementary Figure** 3: Transport properties of bilayer graphene. Transport measurements of a Hall bar and Hall cross produced using bilayer graphene encapsulated in hBN, prepared using our blister cleaning method. a) Resistivity of the sample measured as a function of the back gate voltage at T = 290K and T = 9K. An optical image of the Hall bar is shown in the inset of a). The scale bar is 10$\mu$m. The Hall bar width is 9$\mu$m. The capacitance of the back gate is $C \sim 6.15 \times 10^{-5}\mathrm{F/m^2}$, extracted from a Hall measurement. The mobility reaches $\mu \sim 40\,000\mathrm{cm^2V^{-1}s^{-1}}$ at T = 290K and $\mu \sim 500\,000\mathrm{cm^2V^{-1}s^{-1}}$ at T = 9K. b) Magnetotransport measurements performed on the Hall bar shown in A at T = 9K and B = 2.5T. c) Bend resistance of a Hall cross with arm width $W = 1\mu$m, fabricated from the same heterostructure as the Hall bar in a, measured at T = 9K. An optical image of the Hall cross is shown in the inset. The scale bar is 1$\mu$m. The negative bend resistance indicates $l_m > W$.

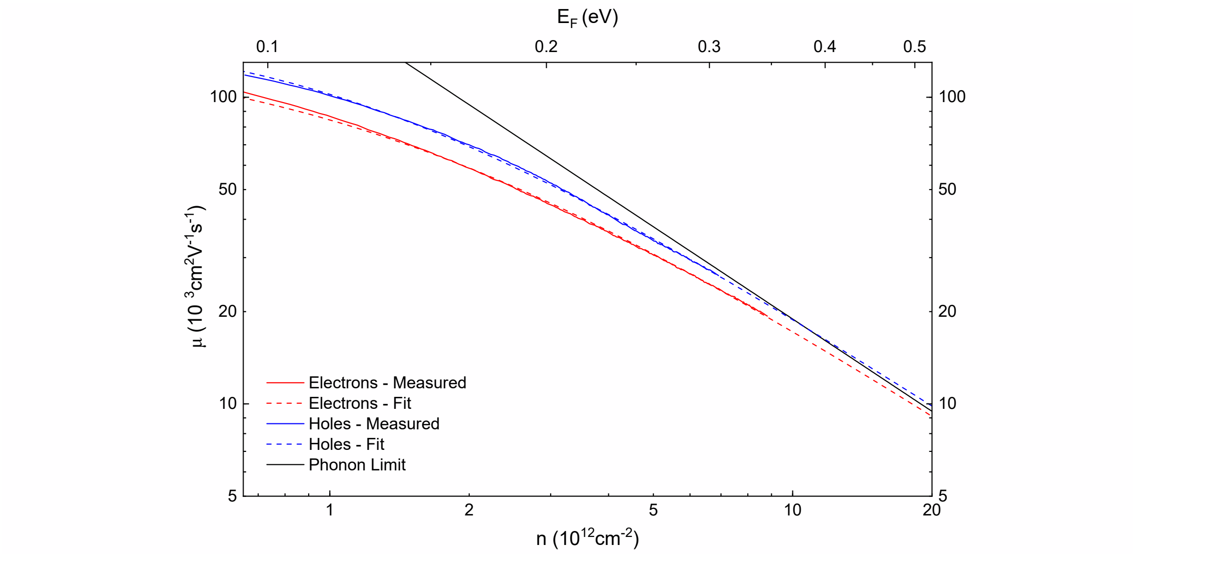


**Supplementary Figure** 4: Mobility as a function of charge carrier density for SLG encapsulated in hBN. The data corresponds to sample S16 in Supplementary Table 1. The data are fit using $\sigma^{-1} = (ne\mu_L + \sigma_0)^{-1} + \rho_s$, which for electrons(holes) yields $\mu_L = 214000(140700)\mathrm{cm^2V^{-1}s^{-1}}$ and $\rho_s = 30.3(32.1)\Omega$. The mobility limit resulting from electron-phonon scattering $\mu_{e\text{-}ph} = 1/(ne\rho_{e\text{-}ph})$ is calculated assuming $\rho_{e\text{-}ph} \sim 33\Omega$ following Ref.[1].

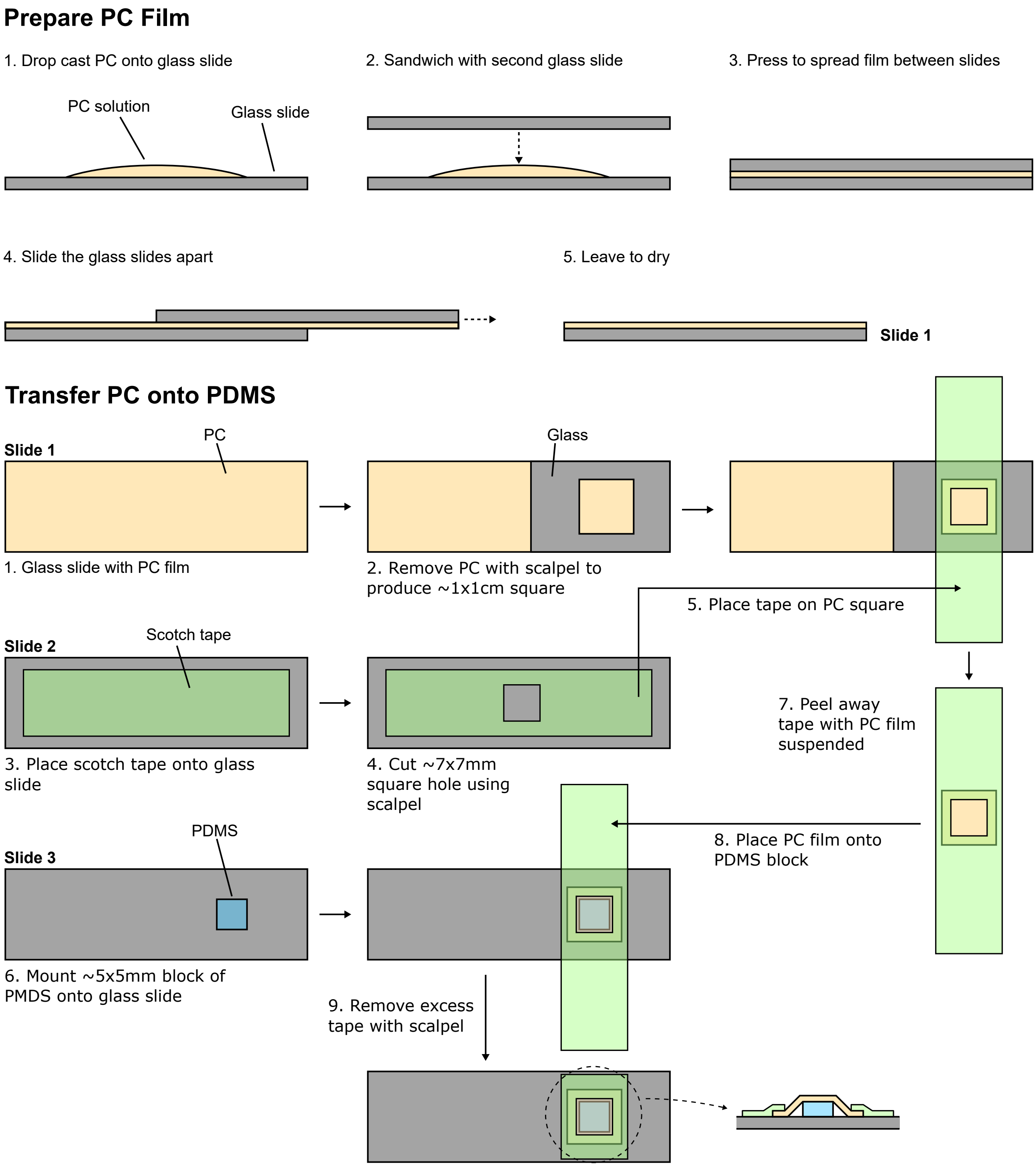


**Supplementary Figure** 5: Preparation of the polycarbonate stamp. The polycarbonate (PC) solution is prepared by dissolving 5% by weight polycarbonate in chloroform. The method for preparing the transfer stamp is outlined in the above figure. The PC is drop cast onto a glass slide using a pipette (typically 10-20 drops), and a second slide is then used to sandwich and spread the solution between the two slides. The slides are immediately slid apart, and left to allow the chloroform to evaporate. Excess PC is then removed by a scalpel to define a $\sim 1 \times 1$cm square of PC. A window is cut into a piece of scotch tape, which is then used to pick up the PC from the glass slide. The tape is then used to place the PC onto a block of PDMS, and excess tape is removed again using a scalpel. For the completed stamp, the PC is held in place on the PDMS by the scotch tape. During the final step of the cleaning process, where the PC is brought into contact with the Si + $SiO_2$ surface and the temperature is raised to 180°, the edges of the PC tear, releasing it from the scotch tape, allowing the stamp to be withdrawn while the PC remains on the Si + $SiO_2$ surface.

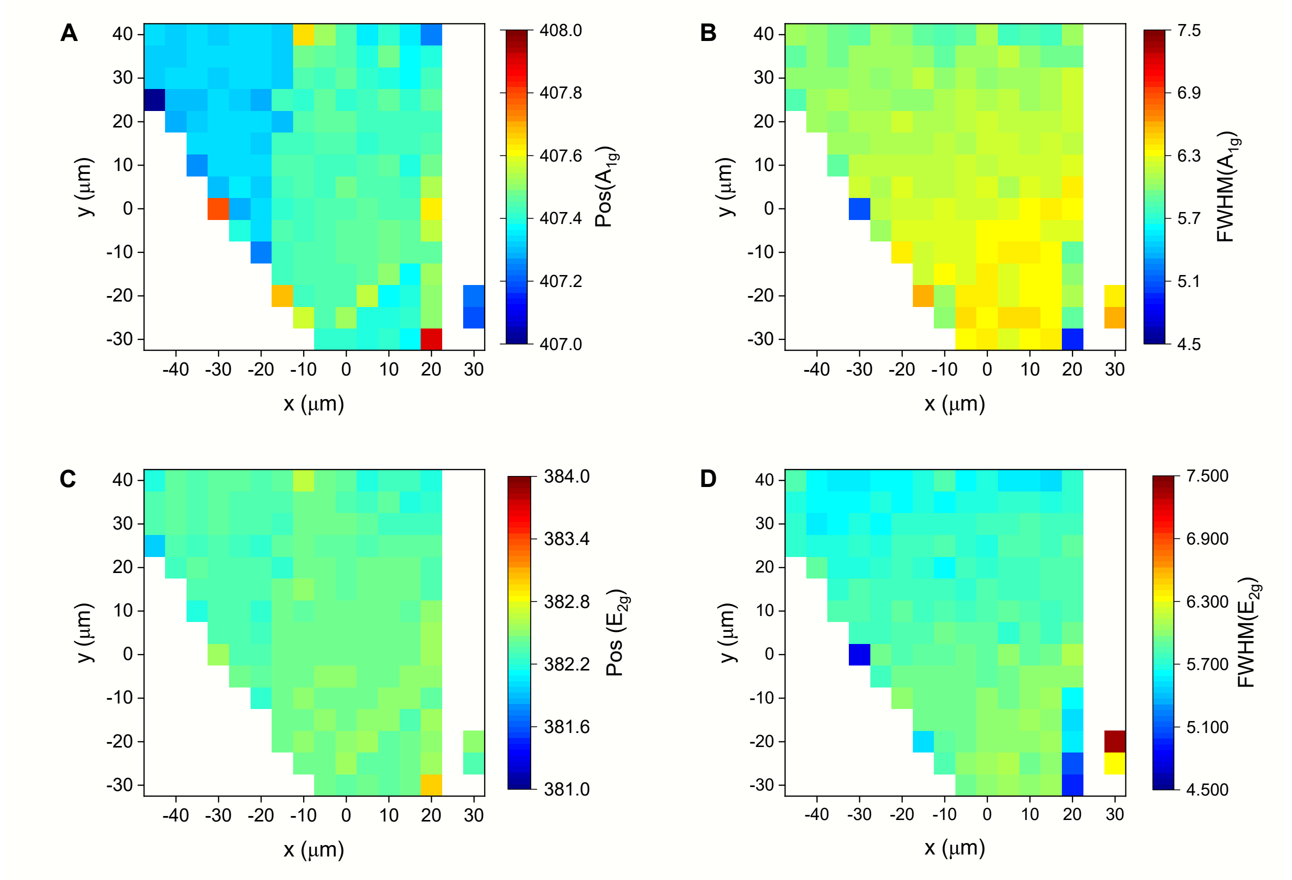


**Supplementary Figure** 6: Raman mapping of an hBN/SLG/$MoS_2$ sample, showing peaks associated with $MoS_2$. The sample is the same as that in Figures 3d-f in the main text. a) Pos($A_{1g}$). b) FWHM($A_{1g}$). c) Pos($E_{2g}$). d) FWHM($E_{2g}$.)

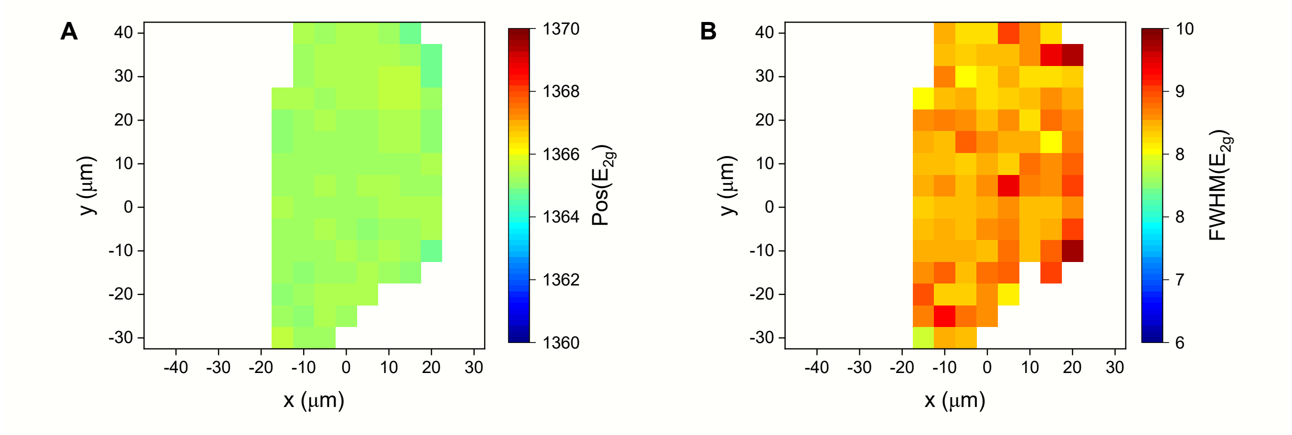


**Supplementary Figure** 7: Raman mapping of an hBN/SLG/$MoS_2$ sample, showing peaks associated with hBN. The sample is the same as that in Figures 3d-f in the main text. a) Pos($E_{2g}$). b) FWHM($E_{2g}$)

# Supplementary Table

## Summary of the electrical transport and Raman measurements of encapsulated SLG Hall bars produced using the blister cleaning method

| **Sample** | | | | **Raman** | | | | | | **Transport** | |
|---|---|---|---|---|---|---|---|---|---|---|---|
| Hall Bar | $W$ | $L$ | $t_{\text{b-hBN}}$ | Pos(2D) | Pos(G) | FWHM(2D) | FWHM(G) | I(2D)/I(G) | A(2D)/A(G) | $\mu_{290\text{K}}$ | $\mu_{9\text{K}}$ |
| S1 | 24 | 21 | 156 | 2689.9 | 1582.0 | 17.3 | 12.8 | 9.4 | 12.7 | 180 | 1800 |
| S2 | 18 | 18 | 156 | 2687.8 | 1580.8 | 16.5 | 14.5 | 12.3 | 14.0 | 170 | 1300 |
| S3 | 7.5 | 7.5 | 176 | 2688.5 | 1581.1 | 16.8 | 14.8 | 17.0 | 19.3 | 160 | 1000 |
| S4 | 17 | 16 | 117 | 2692.7 | 1583.3 | 18.5 | 11.0 | 3.4 | 5.6 | – | – |
| S5 | 22 | 22 | 58 | 2693.1 | 1582.7 | 17.6 | 13.4 | 16.4 | 21.6 | 130 | 400 |
| S6 | 16 | 22 | 93 | 2688.6 | 1581.2 | 17.9 | 12.4 | 6.5 | 9.4 | 180 | 950 |
| S7* | 9 | 11 | 147 | 2691.1 | 1582.2 | 17.2 | 13.4 | 8.6 | 11.0 | 150 | 1300 |
| S8 | 18 | 18 | 62 | 2692.8 | 1582.8 | 17.3 | 14.3 | 9.1 | 10.9 | 180 | – |
| S9 | 15 | 15 | 47 | 2690.6 | 1581.9 | 17.6 | 14.0 | 12.8 | 16.0 | 120 | 1800 |
| S10 | 16 | 22 | 143 | 2690.9 | 1582.1 | 17.1 | 14.0 | 8.9 | 10.8 | 160 | 750 |
| S11 | 12 | 13 | 71 | 2691.5 | 1582.0 | 17.4 | 12.5 | 14.6 | 20.2 | 180 | 800 |
| S12 | 12 | 15 | 66 | 2690.8 | 1581.9 | 17.0 | 12.7 | 19.9 | 26.7 | 160 | 800 |
| S13 | 10 | 11.5 | 93 | 2686.6 | 1580.4 | 17.6 | 12.5 | 7.3 | 10.3 | 140 | 970 |
| S14* | 3 | 4 | 119 | 2690.5 | 1582.1 | 20.1 | 12.9 | 4.8 | 7.6 | 165 | 200 |
| S15* | 2.5 | 4 | 16 | 2693.0 | 1582.5 | 17.7 | 13.2 | 10.4 | 13.9 | 125 | 390 |
| S16 | 3 | 4 | 31 | 2692.6 | 1582.4 | 16.4 | 14.7 | 14.8 | 16.6 | 140 | – |
| S17 | 8 | 7.75 | 2.7 | 2693.9 | 1583.4 | 17.0 | 12.2 | 7.4 | 8.5 | 60 | – |
| S18 | 8 | 7.75 | 6 | 2693.5 | 1583.0 | 16.9 | 12.3 | 7.2 | 9.9 | 75 | – |

Supplementary Table 1: Data for eighteen different Hall bars (S1-S18). *denotes samples produced with graphene exposed to PMMA/Acetone/IPA before encapsulation. $W$: Hall bar channel width. $L$: Hall bar voltage probe arm separation. $t_{\text{b-hBN}}$: Thickness of the bottom hBN flake of the heterostructure (nm). Pos(2D): Position of the 2D peak ($\text{cm}^{-1}$). Pos(G): Position of the G peak ($\text{cm}^{-1}$). FWHM(2D): full width half maximum of the 2D peak ($\text{cm}^{-1}$). FWHM(G): Full width half maximum of the G peak ($\text{cm}^{-1}$). I(2D)/I(G): 2D to G peak intensity ratio. A(2D)/A(G): 2D to G peak area ratio. In the case Raman mapping has been performed on the sample (S1−S10) the Raman parameters are the average value mapped across the sample. For the other samples (S11−S18) the parameters are extracted from a single spectra taken at the center of the sample. $\mu_{290\text{K}}$: Mobility of the Hall bar measured at T = 290K ($10^3\text{cm}^2\text{V}^{-1}\text{s}^{-1}$). $\mu_{9\text{K}}$: Mobility of the Hall bar measured at T = 9K ($10^3\text{cm}^2\text{V}^{-1}\text{s}^{-1}$). The given values of $\mu$ are the peak values of $\mu(n)$ measured for each sample. When calculating the average RT $\mu \sim 160000\text{cm}^2\text{V}^{-1}\text{s}^{-1}$ quoted in the main text we consider only samples S1−S16 where $t_{\text{b-hBN}} > 10$nm.

## Supplementary References


[1] Park, C. H. *et al.* Electron-Phonon Interactions and the Intrinsic Electrical Resistivity of Graphene. *Nano Letters* **14**, 1113-1119 (2014).
