# Supplementary material for: Cleaning interfaces in layered materials heterostructures
Source: Nat Commun. 2018 Dec 19;9:5387. doi: 10.1038/s41467-018-07558-3 (PMC6300598; doi:10.1038/s41467-018-07558-3)
Supplement: Supplementary file 2 — Description of Additional Supplementary Files [file 41467_2018_7558_MOESM2_ESM.pdf]

Description of Additional Supplementary Files

Supplementary Movie 1

Blister cleaning of a hBN/SLG/hBN heterostructure. The sample corresponds to that shown in Figure 1 H-M in the main text. Blisters are pushed by the advancing CF. When blisters meet other blisters they aggregate. Blisters are pushed until they reach the edge of the heterostructure when they are eliminated. One blister remains stuck within the heterostructure, pinned by a wrinkle in the hBN.

Supplementary Movie 2

Blister cleaning of a second hBN/SLG/hBN heterostructure. An optical bright field image, AFM scan, and spatial map of FWHM(2D) of the sample are shown in Supplementary Figure 2 C, G, and K. A number of blisters can be observed becoming pinned when the reach the edge of the SLG region. The interior of the graphene flake (an area of ~100µm ×40µm) is entirely cleaned apart from a single residual blister (see Supplementary Figure 2G).

Supplementary Movie 3

Continuous blister manipulation. The sample is the same as that shown in Supplementary Movie 2. A blister is shown to be moved first in one direction, and then in the opposite direction.

Supplementary Movie 4

Blister cleaning of a MoS_2/hBN heterostructure. The sample corresponds to that shown in Figure 3A-C in the main text. The majority of the blisters are pushed to the bottom of the MoS_2 flake.

Supplementary Movie 5

Blister cleaning of a hBN/SLG/MoS_2 heterostructure. The sample corresponds to that shown in Figure 3D-F in the main text. The majority of blisters are pushed to the bottom edge of the SLG flake where they become pinned (shown by the dashed circle in Figure 3D and 3F). A single large blister remains at the centre of the sample after cleaning.
